# Criterion validity and sensitivity to change of the Early Rehabilitation Index (ERI): results from a German multi-center study

**DOI:** 10.1186/s13104-016-2154-8

**Published:** 2016-07-21

**Authors:** Jens D. Rollnik, M. Bertram, C. Bucka, M. Hartwich, M. Jöbges, G. Ketter, B. Leineweber, M. Mertl-Rötzer, D. A. Nowak, T. Platz, K. Scheidtmann, R. Thomas, F. von Rosen, C. W. Wallesch, H. Woldag, P. Peschel, J. Mehrholz, M. Pohl

**Affiliations:** Institute for Neurorehabilitation Research (InFo), BDH-Klinik Hessisch Oldendorf, Associated Institute of Hannover Medical School (MHH), Greitstr. 18-28, Hessisch Oldendorf, 31840 Germany; Kliniken Schmieder Heidelberg, Heidelberg, Germany; Neurologische Klinik Westend, Bad Wildungen, Germany; Asklepios Schlossberg Klinik Bad König, Bad König, Germany; Brandenburg Klinik Bernau, Bernau bei Berlin, Germany; Neurologisches Rehabilitationszentrum „Godeshöhe“Bonn, Bonn, Germany; Neurologische Klinik GmbH Bad Neustadt, Bad Neustadt, Germany; Schön Klinik Bad Aibling, Bad Aibling, Germany; Helios Klinik Kipfenberg, Kipfenberg, Germany; BDH-Klinik Greifswald, Greifswald, Germany; Hegau-Jugendwerk Gailingen, Gailingen, Germany; Asklepios Kliniken Schildautal Seesen, Seesen, Germany; Schön Klinik Bad Staffelstein, Bad Staffelstein, Germany; BDH-Klinik Elzach, Elzach, Germany; Neurologisches Rehabilitationszentrum Leipzig, Leipzig, Germany; Department of Public Health, University of Dresden, Dresden, Germany; Klinik Bavaria Kreischa, Kreischa, Germany; Klinik Schloss Pulsnitz, Pulsnitz, Germany

**Keywords:** ERI, Early Rehabilitation Index, Early rehabilitation, Validation

## Abstract

**Background:**

Evaluation of functional status is difficult in neurological and neurosurgical early rehabilitation patients. The Early Rehabilitation Index (ERI) was introduced in Germany over 20 years ago, but since then validation studies are lacking. The ERI (range −325 to 0 points) includes highly relevant items including the necessity of intermittent mechanical ventilation or tracheostomy.

**Methods:**

The present paper analyzed data from a German multi-center study, enrolling 754 neurological early rehabilitation patients. Together with ERI, Barthel Index (BI), Glasgow Coma Scale (GCS), Glasgow Outcome Score Extended, Coma Remission Scale (CRS), Functional Ambulation Categories and length of stay were obtained.

**Results:**

ERI showed significant improvements from admission to discharge (p < 0.001). In addition, there were significant correlations of the ERI upon admission and at discharge with BI, CRS and GCS.

**Conclusions:**

Evaluation of our study data suggest that the ERI may be used as a valid assessment instrument for neurological and neurosurgical early rehabilitation patients.

## Background

Treatment facilities for neurological and neurosurgical early rehabilitation in Germany focus on severely impaired patients, immediately after acute-care hospital treatment [1, 2]. Early rehabilitation facilities carry on intensive and intermediate care treatment including weaning from mechanical ventilation, management of tracheotomy tube and other medical devices [2, 3]. Parallel to intensive care, rehabilitation is commenced in order to improve disturbances of consciousness, swallowing, mobility, coordination and cognition.

Early rehabilitation patients are characterized by high morbidity and frequent colonization with multi-drug resistant (MDR) bacteria [4, 5]. Depending on the diagnosis, prognosis may be poor, in particular among hypoxic brain damage patients [6].

It is difficult to assess neurological early rehabilitation patients and their outcome. Traditional scales including the Glasgow Coma Scale (GCS) [7] or Coma Remission Scale (CRS) [8] focus solely on disorders of consciousness. Other well established assessments such as the Barthel Index (BI) [9] are highly relevant with respect to activities of daily living (ADL). However, a floor effect is commonly seen with the BI since most of the early rehabilitation patients are completely dependent on nursing throughout the whole rehabilitation process. Another scale, called early functional abilities (EFA) [10] is common among early rehabilitation facilities, but there exists only scant data on its validation.

More than 20 years ago, the Early Rehabilitation Barthel Index (ERBI) was introduced into clinical practice as an extension of the traditional BI [11]. The ERBI is a sum score of BI and Early Rehabilitation Index (ERI). The ERI comprises highly relevant items to assess neurological and neurosurgical early rehabilitation patients, such as intensive care monitoring, tracheostoma management, mechanical ventilation or swallowing disorders, see Table 1. Each ERI-item—if applicable—has a negative value of −50 or −25 points. To compute the ERBI, ERI sum (0 to −325 points) is subtracted from the BI (0 to +100 points), resulting in an ERBI ranging from −325 to 100 points. While ERBI has even entered the German DRG- (Diagnosis Related Groups) system to define the early rehabilitation procedure 8-552 [12], data on its criterion validity is scarce and the definition of ERI items is often inprecise [13]. Two German expert groups have published criteria for operationalization of the ERI yielding broad consent [13, 14].

**Table 1:** Early Rehabilitation Index (ERI) [11, 19]

Item applicable	Yes	No
1. Intensive medical monitoring	−50	0
2. Tracheostoma requiring special treatment (suctioning)	−50	0
3. Intermittent (or continuous) mechanical ventilation	−50	0
4. Confusional state requiring special supervision	−50	0
5. Behavioral disturbances requiring special care (patient poses a risk to himself or his environment)	−50	0
6. Severe communication deficits	−25	0
7. Swallowing disorders requiring special supervision	−50	0
Sum score ERI	−325 to 0 points	

The ERI has been used to assess patients suffering from locked-in-syndrome [15], dysphagia [16], traumatic brain injury [17] and hypoxic brain damage [6]. In addition, ERI was applied in some multi-center studies on the outcome of early rehabilitation patients [2, 18].

Another publication demonstrated that patients with a low ERBI have a significantly longer length of stay (LOS) [1]. A one-center study focused on reliability and validity of the ERI in a large sample of neurological and neurosurgical early rehabilitation patients [19]. In one sample (n = 1,669), estimates of morbidity and LOS were compared with ERI categories [19]. In addition, inter-rater reliability (nurses vs. physicians) was examined in a second sample of 273 patients [19]. Patients with low ERBI had a significantly longer LOS than those with high ERBI values (p < 0.001) [19]. Further, parameters of morbidity (patient clinical complexity level, number of co-diagnoses) were significantly higher in a subgroup with lower ERBI [19]. Inter-rater reliability was high (r = 0.849, p < 0.001) [19]. The findings suggested that the ERI might be a reliable and valid scale to assess early neurological rehabilitation patients [19].

The present paper is based upon data from a 2014 German multi-center study on neurological and neurosurgical early rehabilitation [2]. The main focus was to examine the criterion validity of the ERI [19].

## Methods

The prospective multi-center study enrolled data of 754 patients admitted to 16 German neurological early rehabilitation centers in 2014 [2]. Inclusion and exclusion criteria have been published in a previous paper [2].

Age, gender, main diagnosis, medical devices, Glasgow Coma Scale (GCS) [7], Coma Remission Scale (CRS) [8], Barthel Index (BI) [9], Early Rehabilitation Index (ERI) [19] and FAC (Functional Ambulation Categories) [20] upon admission and at discharge were documented. FAC categorizes patients according to basic motor skills necessary for functional ambulation ranging from 0 (“nonfunctional”) to 5 (“independent, level and non-level surfaces” [20]). In addition, Glasgow Outcome Scale-Extended (GOSE) [21] was obtained at discharge.

In line with previous studies [6, 22], patients were assigned to a dichomotous outcome: poor (BI at discharge <50) vs. good (BI at discharge ≥50). According to this definition, 630 (83.6 %) patients had a poor and only 124 (16.4 %) a good outcome at the end of the early rehabilitation. It has to be pointed out that even patients belonging to the poor outcome group might have a good functional status, finally, after subsequent rehabilitation [2].

Statistics: Since main variables including ERI upon admission and at discharge were not normally distributed, non-parametric testing was performed. Statistical analyses included χ^2^-for categorical variables, Mann–Whitney-U- and Kruskal–Wallis-tests for independent, Wilcoxon-tests for paired samples. In addition, Spearman-Rho correlations were computed. Differences were regarded as significant with p < 0.05. Nonetheless, in the results section, mean values and standard deviations are displayed. Interquartile ranges (Q3–Q1) were computed for the main clinical scales. For statistical analyses, SPSS^®^ (version 21) was used.

Ethics: The study required no ethical approval since it was a data analysis, relying on measurements and data acquisition applied as part of routine care in neurological early rehabilitation. The need for ethical approval has been deemed unnecessary according to national legislation. Patient data were anonymized prior to analysis.

## Results

Mean age of the whole study sample was 68.0 ± 14.8 years, 297 (39.4 %) subjects were female, 457 (60.6 %) male.

Primary diagnoses can be found in Table 2 demonstrating that most of the early rehabilitation patients were suffering from ischemic stroke (31.7 %) or intracranial hemorrhage (20.4 %).

**Table 2:** Main diagnoses of the study sample [2]

	n	%
Ischemic stroke	239	31.7
Intracranial hemorrhage	154	20.4
Polyneuropathy or other peripheral nerve impairment	131	17.4
Traumatic brain injury	87	11.5
Hypoxia	47	6.2
Spinal cord injury	28	3.7
Brain tumor	21	2.8
Other main diagnosis	47	6.2
Sum	754	100

Analyzing the changes from admission to discharge, all assessments (ERI, BI, CRS, GCS) showed significant improvements (p < 0.001), see Table 3. Interquartile ranges for the clinical scales may be found in Table 4. Significant changes were also demonstrated with FAC (χ^2^-test, p < 0.001), see Table 5. GOSE revealed that most patients were still severely disabled at discharge from early rehabilitation, mortality was 9.5 %, see Table 6.

**Table 3:** Mean values and standard deviations (in brackets) upon admission and at discharge [2]

	Admission	Discharge	*p* value*
Barthel Index (BI) [0 to 100]	8.5 (11.3)	25.0 (22.8)	<0.001
Early Rehabilitation Index (ERI) [−325 to 0]	−112.5 (85.0)	−55.1 (66.1)	<0.001
Coma Remission Scale (CRS) [0 to 24]	18.3 (7.4)	19.9 (7.4)	<0.001
Glasgow Coma Scale (GCS) [3 to 15]	11.7 (3.6)	12.7 (3.8)	<0.001

* Wilcoxon test

**Table 4:** Interquartile ranges (Q3–Q1) for the variables upon admission and at discharge

	Admission	Discharge
Barthel Index (BI) [0 to 100]	15	35
Early Rehabilitation Index (ERI) [−325 to 0]	125	100
Coma Remission Scale (CRS) [0 to 24]	10	4
Glasgow Coma Scale (GCS) [3 to 15]	6	3

**Table 5:** FAC on admision and at discharge [2]

FAC on admission	FAC at discharge						Sum
	0	1	2	3	4	5	
0	418	76	73	50	33	3	653
1	3	9	18	17	3	1	51
2	1	0	6	6	5	1	19
3	2	0	0	1	4	4	11
4	0	0	0	0	0	1	1
5	0	0	0	0	0	0	0
Sum	424	85	97	74	45	10	735

**Table 6:** Glasgow Outcome Scale Extended (GOSE) at discharge [2]

GOSE	Sum
1. Death	72 (9.5 %)
2. Vegetative state	57 (7.6 %)
3. Lower severe disability	287 (38.1 %)
4. Upper severe disability	315 (41.8 %)
5. Lower moderate disability	23 (3.1 %)
6. Upper moderate disability	0
7. Lower good recovery	0
8. Upper good recovery	0
Sum	754 (100 %)

Spearman-Rho correlations showed a significant negative correlation between changes in ERI and age, suggesting that smaller improvements were found the older the patients were, see Table 7. However, the correlation coefficient was small. In addition, ERI upon admission, at discharge and ΛERI (discharge minus admission) correlated significantly and positively with GCS, CRS and BI upon admission and at discharge (Table 7). LOS was longer when ERI was lower (admission: r_s_ = −0.367, p < 0.001; discharge: r_s_ = −0.152, p < 0.001).

**Table 7:** Bivariate Spearman-Rho correlations

Variable	ERI upon admission (−325 to 0)	ERI at discharge (−325 to 0)	ΛERI (admission-discharge)
BI upon admission (0–100)			
r	0.575**	0.304**	−0.349**
p	0.000	0.000	0.000
n	754	754	754
BI at discharge (0–100)			
r	0.262**	0.502**	0.154**
p	0.000	0.000	0.000
n	754	754	754
ΛBI			
r	0.060	0.442**	0.320**
p	0.102	0.000	0.000
n	754	754	754
GCS upon admission (3–12)			
r	0.466**	0.331**	−0.243**
p	0.000	0.000	0.000
n	715	715	715
GCS at discharge (3–12)			
r	0.353**	0.553**	0.086*
p	0.000	0.000	0.025
n	689	689	689
ΛGCS (discharge-admission)			
r	−0.202**	0.087*	0.349**
p	0.000	0.023	0.000
n	688	688	688
CRS upon admission (0–24)			
r	0.444**	0.331**	−0.213**
p	0.000	0.000	0.000
n	689	689	689
CRS at discharge (0–24)			
r	0.330**	0.526**	0.092*
p	0.000	0.000	0.018
n	664	664	664
ΛCRS (discharge-admission)			
r	−0.221**	0.010	0.307**
p	0.000	0.807	0.000
n	663	663	663
GOSE (discharge) (1–8)			
r	0.293**	0.472**	0.093*
p	0.000	0.000	0.011
n	754	754	754
Age (years)			
r	0.075*	0.002	−0.084*
p	0.039	0.967	0.021
n	754	754	754
Disease duration (days prior to admission)			
r	−0.131**	−0.093*	0.019
p	0.000	0.011	0.604
n	749	749	749
Length of stay (days)			
r	−0.367**	10.152**	0.327**
p	0.000	0.000	0.000
n	754	754	754

* p < 0.05. ** p < 0.01, r = Spearman Rho correlation coefficient, p = *p* value, n = sample size

Patients belonging to the good outcome group (BI ≥50) had a significantly better ERI value at discharge (Mann–Whitney-U-test: p < 0.001) and bigger ERI improvements (Mann–Whitney-U-test: p < 0.001) than poor outcome subjects.

Focusing on the three most frequent diagnoses (ischemic stroke, intracranial hemorrhage, polyneuropathy or other peripheral nerve impairment), a Kruskal–Wallis-test revealed that ΛERI differed significantly between the three groups (mean rank: polyneuropathy = 282.99, intracranial hemorrhage = 251.29, ischemic stroke 212.51; p < 0.001) indicating smaller changes when the central nervous system was affected compared to peripheral damage.

## Discussion

Neurological early rehabilitation patients constitute a sample of severely disabled persons. Frequently, they suffer from extreme morbidity, high mortality, low functional status and they may be colonized with MDRs [1–5]. The fact that functional improvements are limited during early rehabilitation is illustrated by the finding that only 16.4 % of our study sample had a BI of at least 50 points (“good outcome”) at discharge from early rehabilitation. Of course, many of the “poor outcome” patients enter subsequent rehabilitation programs where they may further improve their ADL [1, 2, 23].

Since functional improvements are small, traditional ADL scales such as BI are of limited value to assess neurological early rehabilitation patients. Together with other scales like EFA [10], the ERI has been introduced employing highly relevant items like mechanical ventilation or intensive medical monitoring [11]. Even if no BI changes are observed, it is obvious that a patient successfully weaned from ventilation made a fine step forward. There is no doubt that it is much easier to take care of a spontaneously breathing subject than to transfer patients on home ventilation to nursing facilities.

However, only little data is available on the criterion validity, sensitivity to change and inter-rater reliability of the ERI [13, 19]. There is at least one study suggesting that the ERI might be a valid clinical assessment with satisfactory reliability [19].

The present study analyzed data from a multi-center study, enrolling a large number of patients [2]. ERI scores were compared with traditional scales, such as BI, CRS and GCS used to assess severely disabled neurological patients [7–9].

First, all scales proved their sensitivity to change because there were significant changes from admission to discharge. As with BI, GCS and CRS, ERI also showed significant improvements at discharge from early rehabilitation. However, interquartile ranges (IQR) of all main clinical scales (BI, ERI, CRS and CRS) upon admission and at discharge were small indicating that scores were close together and that it was difficult to differentiate between patients. ERI on admission had an IQR of 125, BI of 15. This does not necessarily mean that ERI scores had a larger dispersion than BI because in both scales, the IQR resembles a difference of only three steps (BI: 5, 10, 15; ERI: −125, −75, −50). However, making a sum of BI and ERI (the so-called Early Rehabilitation Barthel Index—ERBI) increases IQR and thus might help to better differentiate patients.

In addition, Spearman-Rho correlations showed a small but significant correlation between changes in ERI and age. This finding suggests that age may have a palpable negative impact on the outcome of these patients. This result is not surprising because age has shown to have a negative correlation with BI changes in many studies on neurological rehabilitation [4].

There was also a small negative correlation between ERI and LOS. This finding is in line with results from a previous study [19]. It may be hypothesized that ERI is an estimate of patients` morbidity contributing to a longer LOS [19].

Furthermore, we found that ERI upon admission, at discharge and ΛERI (discharge minus admission) correlated significantly and positively with GCS, CRS and BI upon admission and at discharge. The fact that ERI scores correlated with well-established scales like GCS, CRS and BI supports the hypothesis that it might be a valid instrument to assess neurological early rehabilitation patients. It has to be pointed out that correlations were moderate indicating that the ERI measures aspects which are not completely covered by other assessments and thus adds additional relevant information.

There was also an interrelation between ERI and outcome (functional independence measured with BI): Patients belonging to the good outcome group (BI ≥50) had a significantly better ERI value at discharge and bigger ERI improvements than poor outcome subjects. This finding also supports the assumption that ERI is of satisfactory criterion validity.

## Conclusions

The findings from our multi-center study suggest that the ERI is a valid scale in assessing neurological and neurosurgical early rehabilitation patients and their rehabilitation progress. There exist significant correlations between ERI and well-established scales like BI, CRS and GCS. ERI, however, adds additional relevant information for the rehabilitation process. The ERI provides additional information on patients` status and improvement and could be used together with the BI as ERBI.

## Abbreviations

ADL: Activities of daily living
BI: Barthel Index
CRS: Coma Remission Scale
DRG: Diagnosis related groups
EFA: Early functional abilities
ERBI: Early Rehabilitation Barthel Index
ERI: Early Rehabilitation Index
FAC: Functional Ambulation Categories
GCS: Glasgow Coma Scale
GOSE: Glasgow Outcome Scale Extended
IQR: Interquartile range
LOS: Length of stay
MDR: Multi-drug resistant
